# Supplementary material for: Structural Insights into the Dimeric Form of Bacillus subtilis RNase Y Using NMR and AlphaFold
Source: Biomolecules. 2022 Dec 1;12(12):1798. doi: 10.3390/biom12121798 (PMC9775385; doi:10.3390/biom12121798)
Supplement: Supplementary file 1 [file biomolecules-12-01798-s001.zip › biomolecules-1959284-supplementary.pdf]

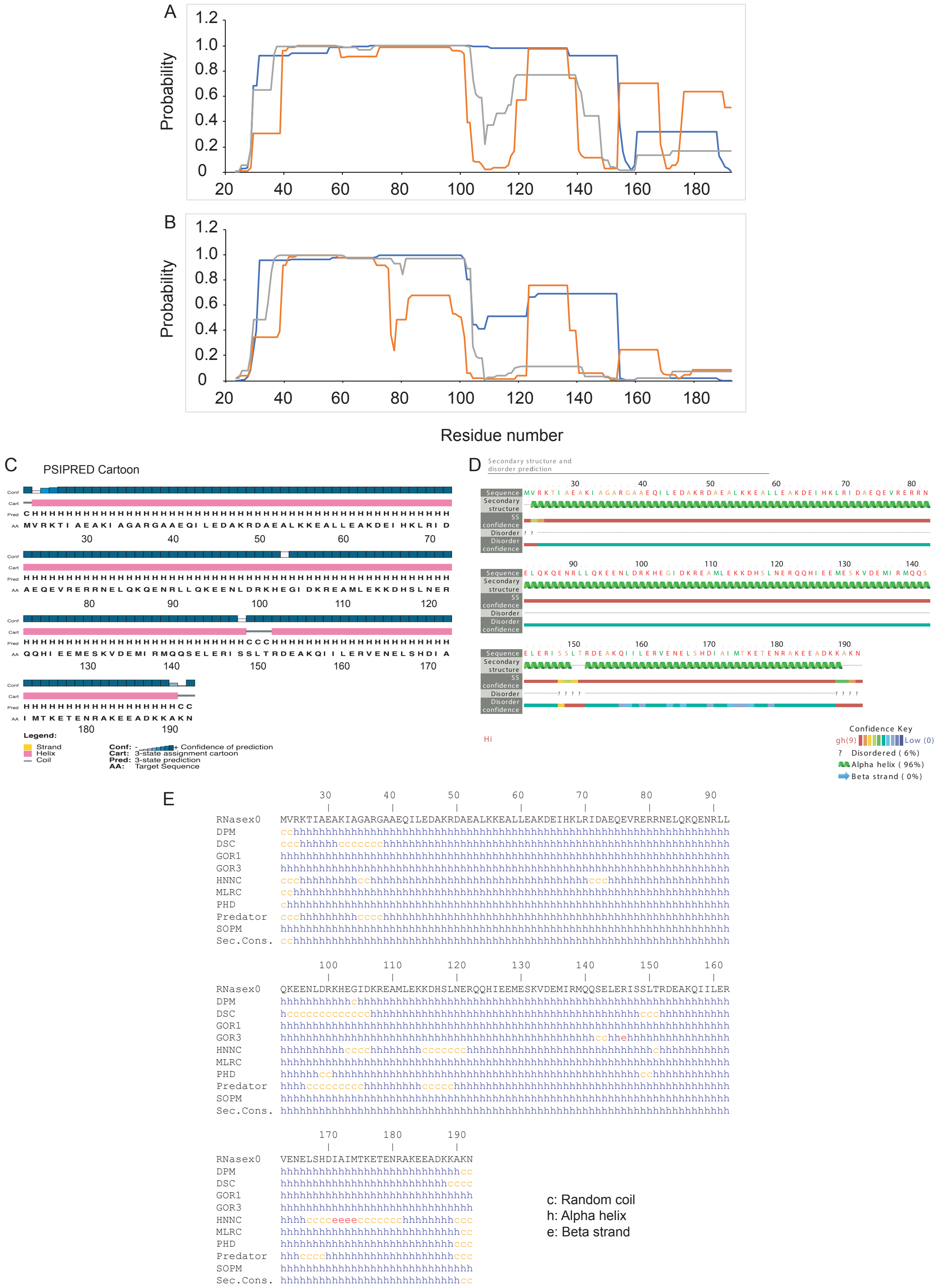

Figure S1: Secondary structure prediction of Nter-BsRNaseY. Coiled-coil prediction using the NPSA_lupas algorithm without (A) or with a weight of 2.5 for positions ‘a’ and ‘d’ in the heptad repeat (B). Secondary structures prediction of Nter-BsRNaseY using PSIPRED (C), PHYRE2 (D) and the consensus secondary structure prediction server NPS@ (E). The amino acids composition indicates that this domain is mostly charged and polar (70%), does not contain any aromatic residue and displays only a few hydrophobic residues.

A

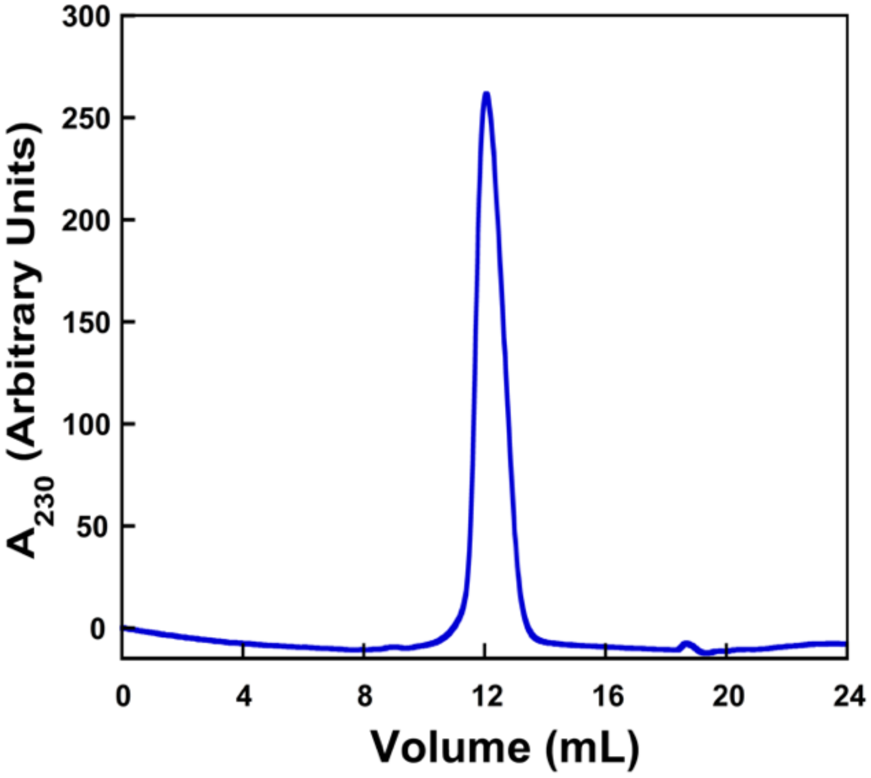

B

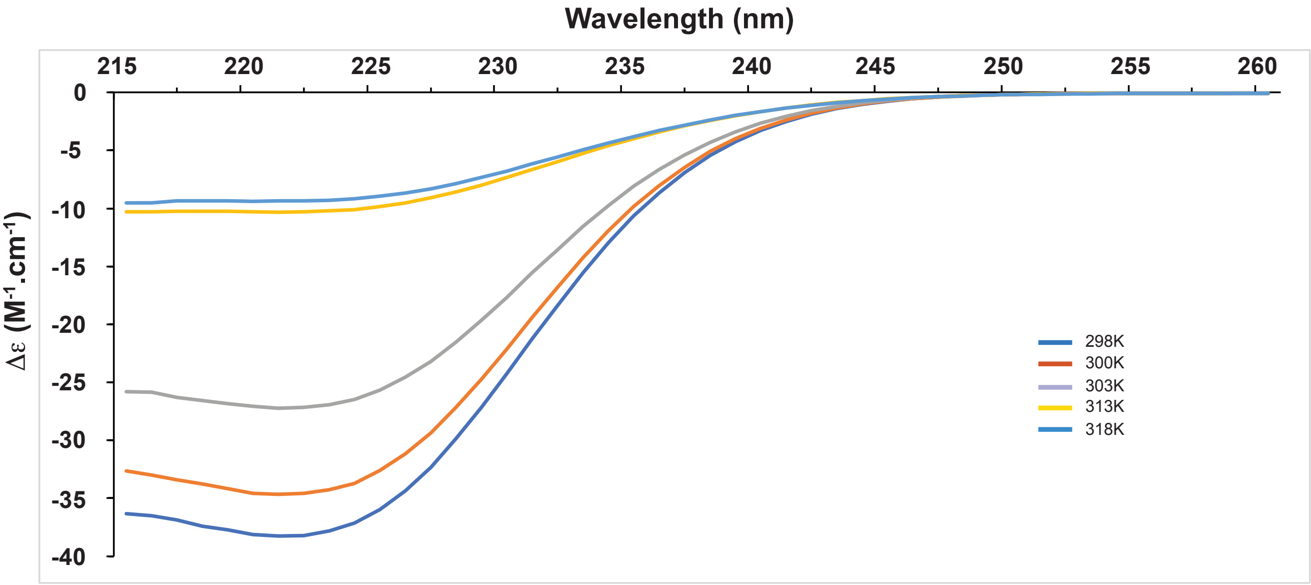

Figure S2. Characterization of Nter-BsRNaseY by SEC and CD. (**A)** Chromatographic profile of Nter-BsRNaseY. As the final step of purification, ^{13}C-^{15}N-^{2}D Nter-BsRNaseY (960 µM), was purified on a Superdex Increase 200 10/300 gel filtration column in 40 mM MES pH 6.8, 200 mM NaCl. **(B)** CD spectrum of Nter-BsRNaseY (485 µM) recorded in 40 mM MES pH 6.2, 200 mM NaCl, 10% D2O as a function of wavelength at various temperatures.

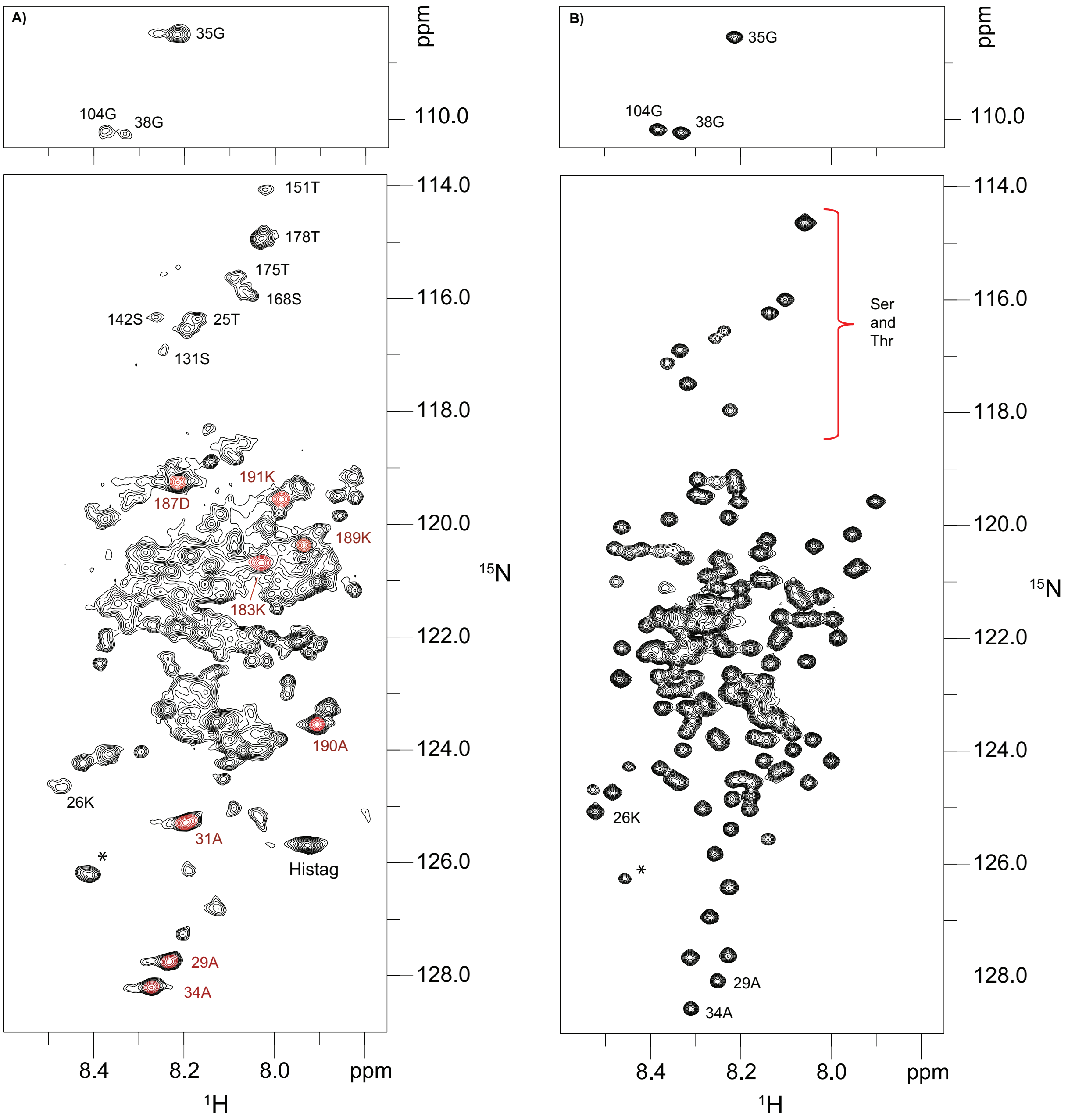

Figure S3: (^{1}H-^{15}N)-HMQC spectra of Nter-BsRNaseY in the absence and presence of urea. The (^{1}H-^{15}N)-HMQC spectra were recorded at 950 MHz and 298K on Nter-BsRNaseY (190 µM) in MES 40 mM pH 6.8, 200 mM NaCl, 5% D_2O in the absence (A) and presence (B) of 6 M of urea. The top panels represent the glycine regions. Only residues 26K, 29A and 34A belonging to the unstructured N-terminus can be assigned unambiguously by comparing the two spectra. * annotates an impurity. Several peaks with strong intensity are highlighted in red. They belong to residues located at the N and C terminus.

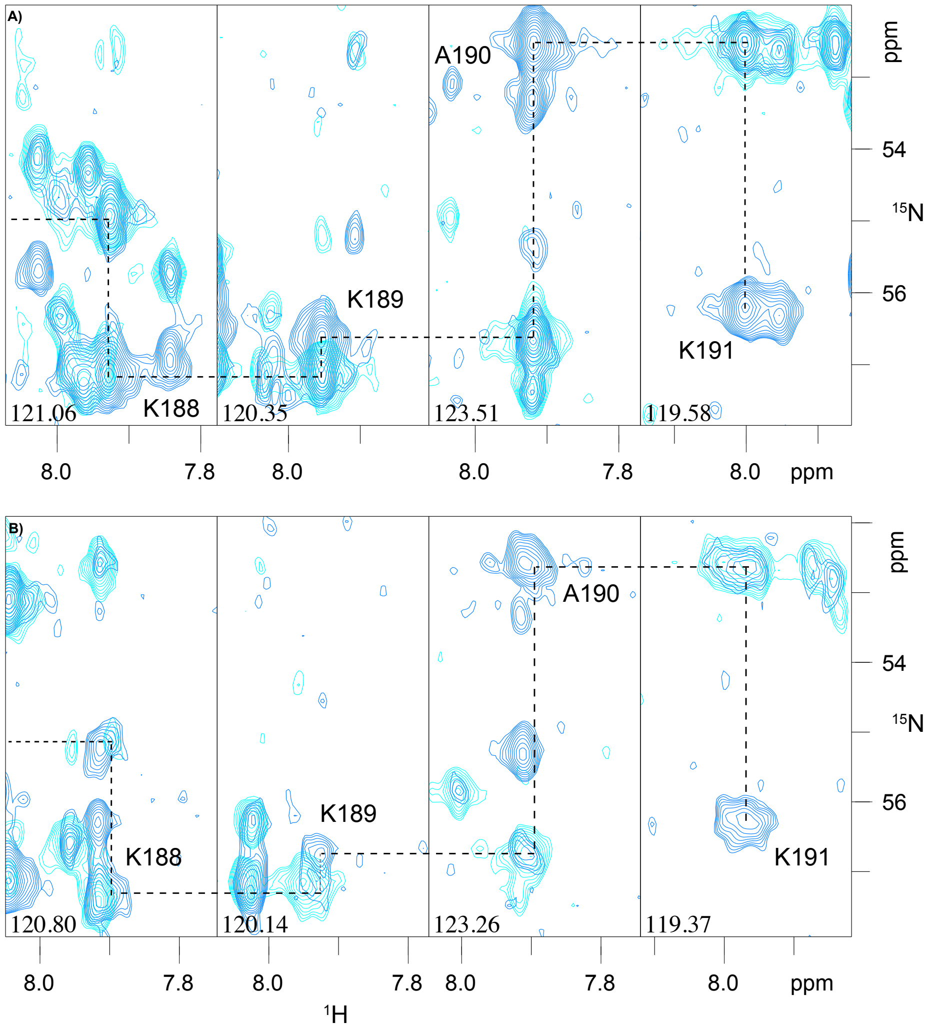

Figure S4: Representative strips from the 3D HNCA (dark blue) and HN(CO)CA (light blue) spectra at 303K, showing the sequential assignment of the K188-K191 segment of Nter-BsRNaseY based on the high intensity peaks (A) and the low intensity peaks (B).

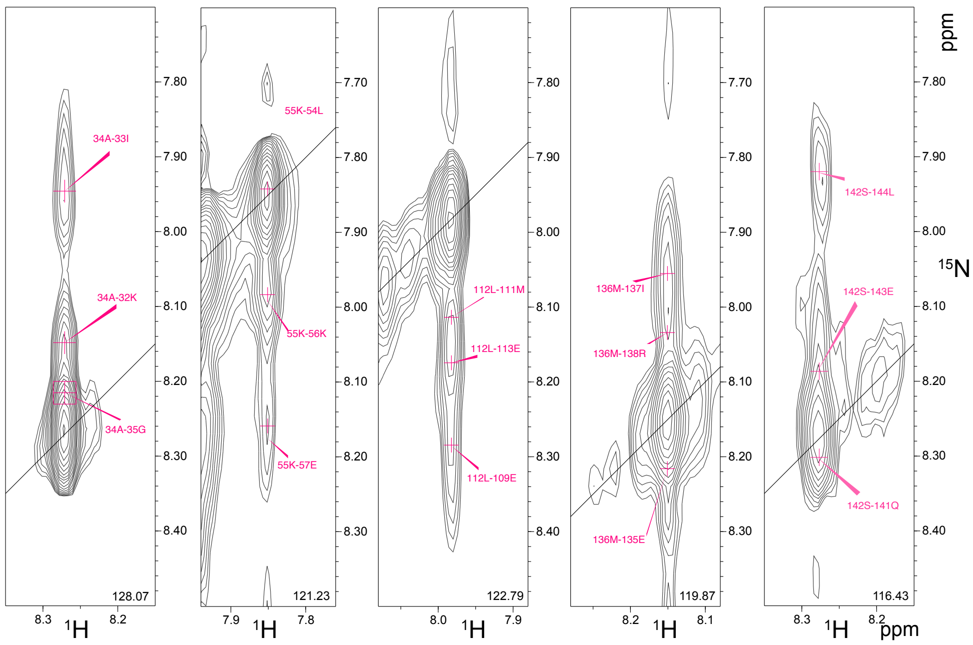

Figure S5: ^{1}H-^{15}N NOESY-HSQC strips showing several examples of NOE peak assignments.

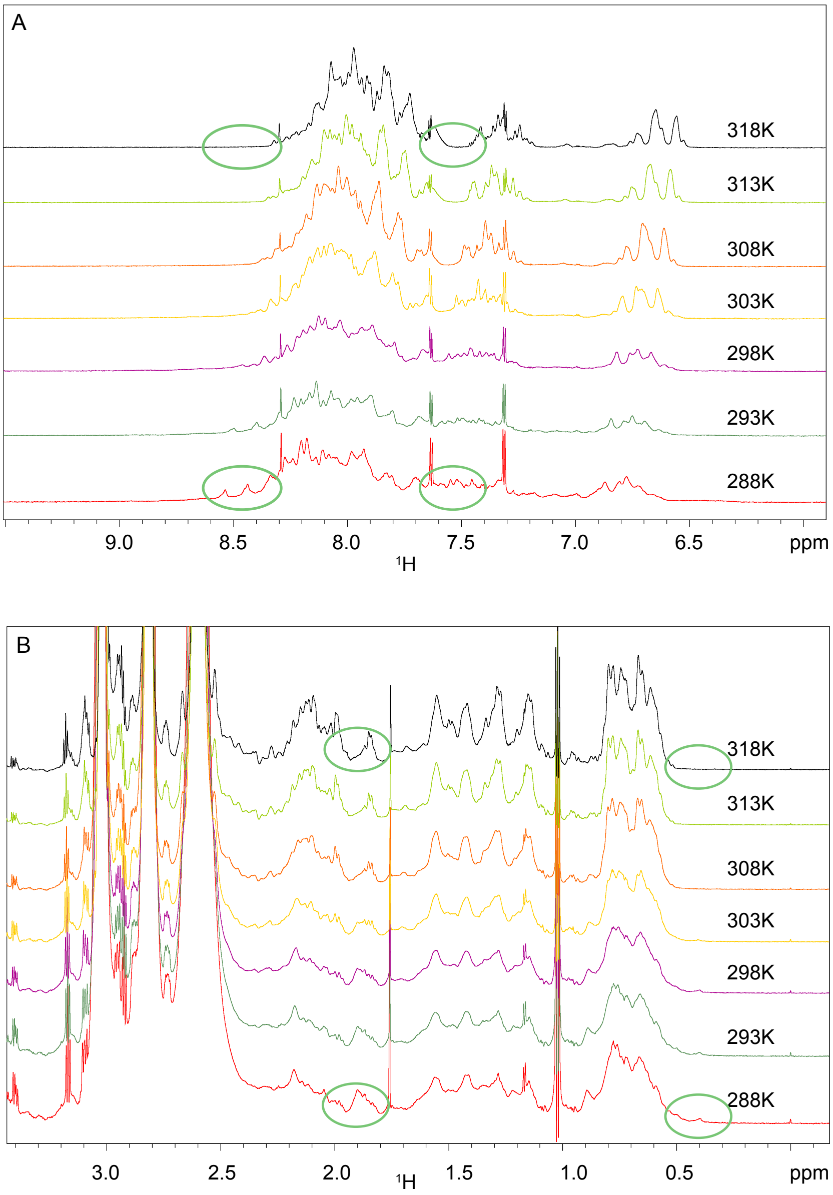

Figure S6: Temperature effect on the amide and aromatic (A) and aliphatic protons (B) of Nter-BsRNAseY. The ^{1}D NMR spectra of Nter-BsRNAseY (970 µM, in 40 mM MES buffer pH 6.8, 200 mM NaCl), were recorded at various temperatures between 288K and 318K at 950 MHz. The green circles highlight the highest differences observed in the ^{1}H chemical shifts at 288K and 318K.

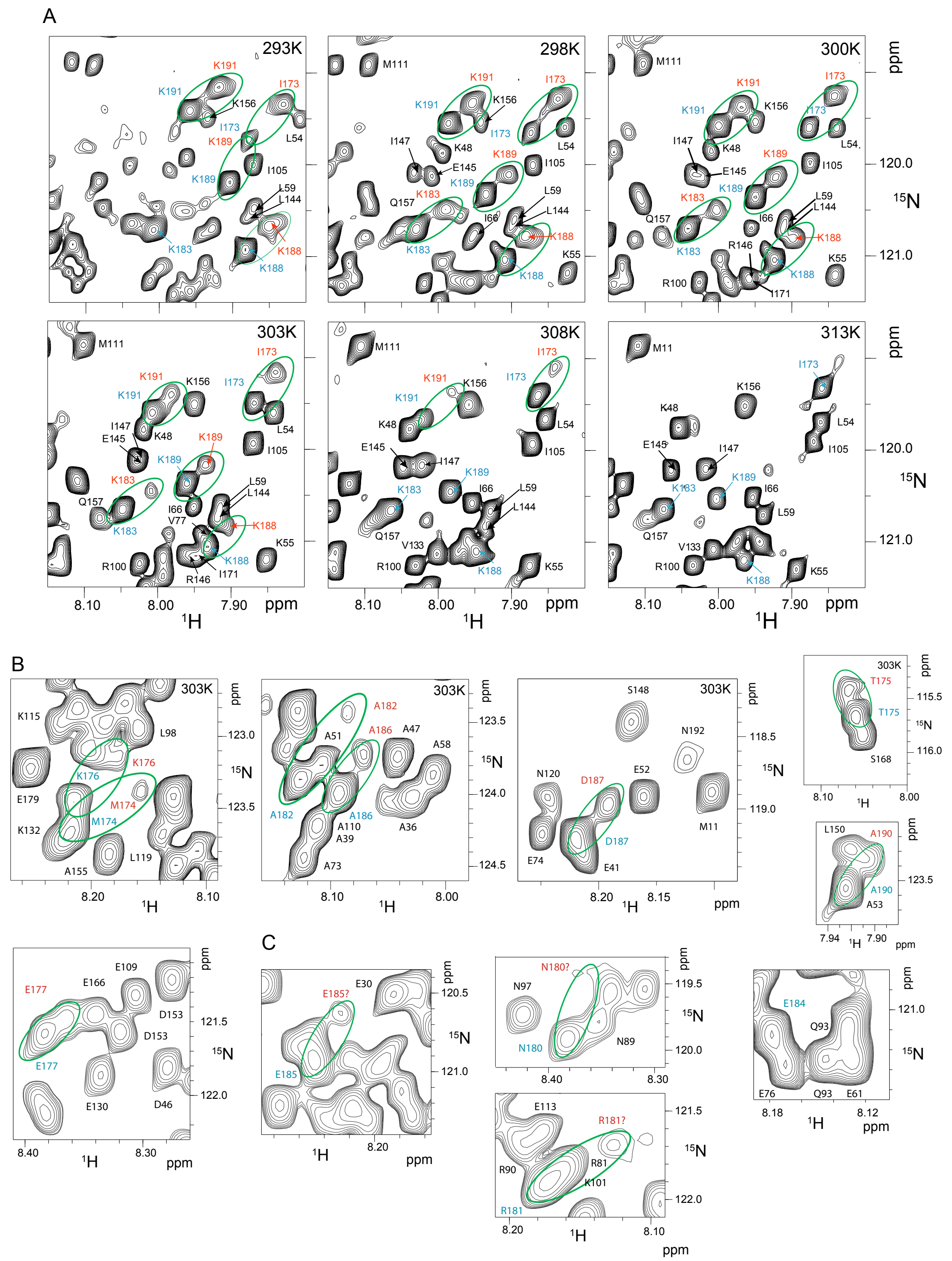

Figure S7: Dependence on the temperature of the duplicated cross-peaks in the ^{1}H-^{15}N BEST-TROSY spectra. (A) Influence of the temperature on the relative intensity of the duplicated cross-peaks of I173, K183, K188, K189, K191. The temperatures are indicated in light blue in the right corner of each panel. (B and C) The duplicated cross-peaks of M174, T175, K176, E177, N180, R181, A182, E185, A186, D187 and A190. The duplicated peaks are marked in blue (high intensity peaks) and red (low intensity peaks). Pairs of split peaks are circled in green. The ? in Panel (C) indicates the likely positions of duplicated peaks for residues N180, R181 and E185, for which the intensity was too low to be detected in the 3D spectra and which could not be used for assignment.

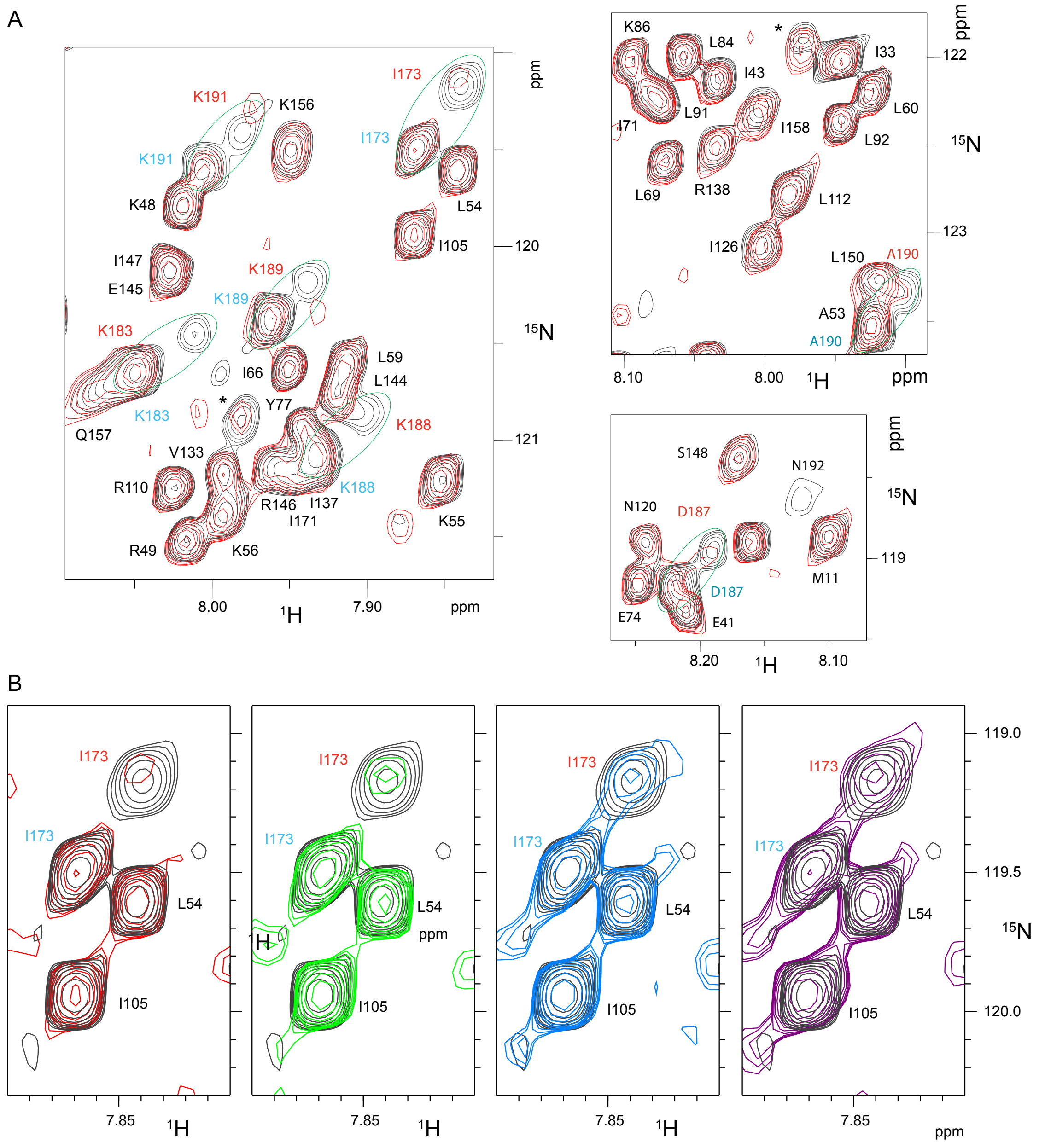

Figure S8: Effect of Nter-BsRNaseY concentration on the ^{1}H-^{15}N BEST-TROSY NMR spectra recorded at 303K. (A) Superposition of the spectra of 970 µM (black) and 50 µM (red) Nter-BsRNaseY showing the disappearance of the duplicated peaks corresponding to residues at the C-terminus (173, 183, 188, 189, 191, contoured in green) at low protein concentration. Pairs of duplicated peaks are circled in green, when the low intensity peaks were detectable. (B) Variation in intensity of one of the duplicated peaks for residue I173, depending on the Nter-BsRNaseY concentration: 50 µM (red), 98 µM (green), 195 µM (light blue), 332 µM (magenta) and 970 µM (black), showing chemical shift variations dependent on protein concentration. The duplicated peak is marked in blue (high intensity peak) and red (low intensity peak).

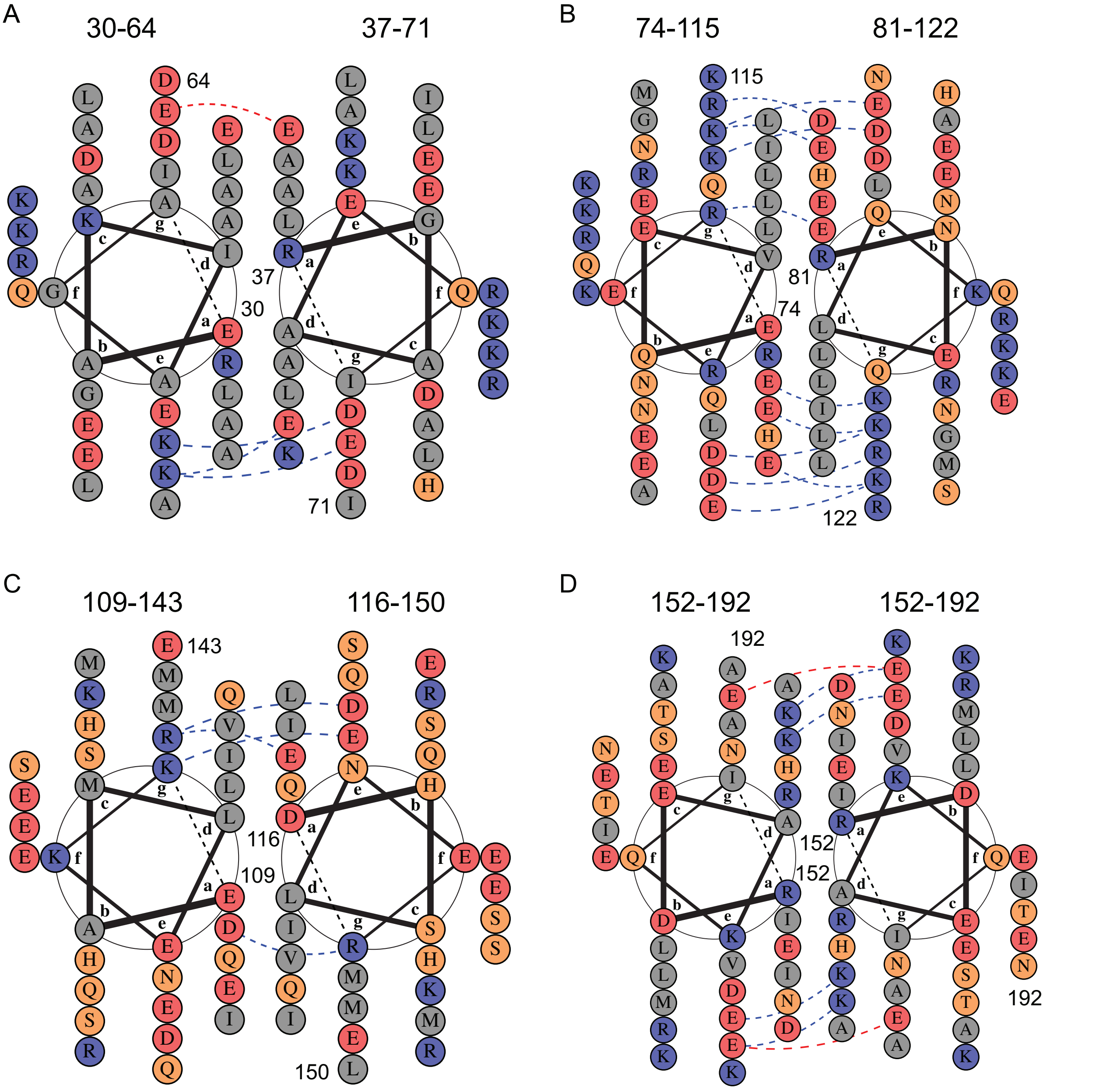

Figure S9: Helical wheel representation of the parallel coiled-coil fold predicted in the best AlphaFold model of the Nter-BsRNaseY dimer. A) residues 30-71, B) residues 74-122, C) residues 109-150, D) residues 152-192. The first and last residue of each segment are labeled. The intermolecular electrostatic interactions between the coils are displayed by dashed lines, with the favorable and unfavorable ionic interactions in blue and red respectively. ‘a’, ‘b’, ‘c’, ‘d’, ‘e’, ‘f’, ‘g’ indicate the positions forming the heptad repeats, which are at the basis of the coiled-coil structure. The hydrophobic residues are colored in grey, the negatively charged residues in red, the positively charged residues in blue and those that are susceptible to form hydrogen bonds in orange.

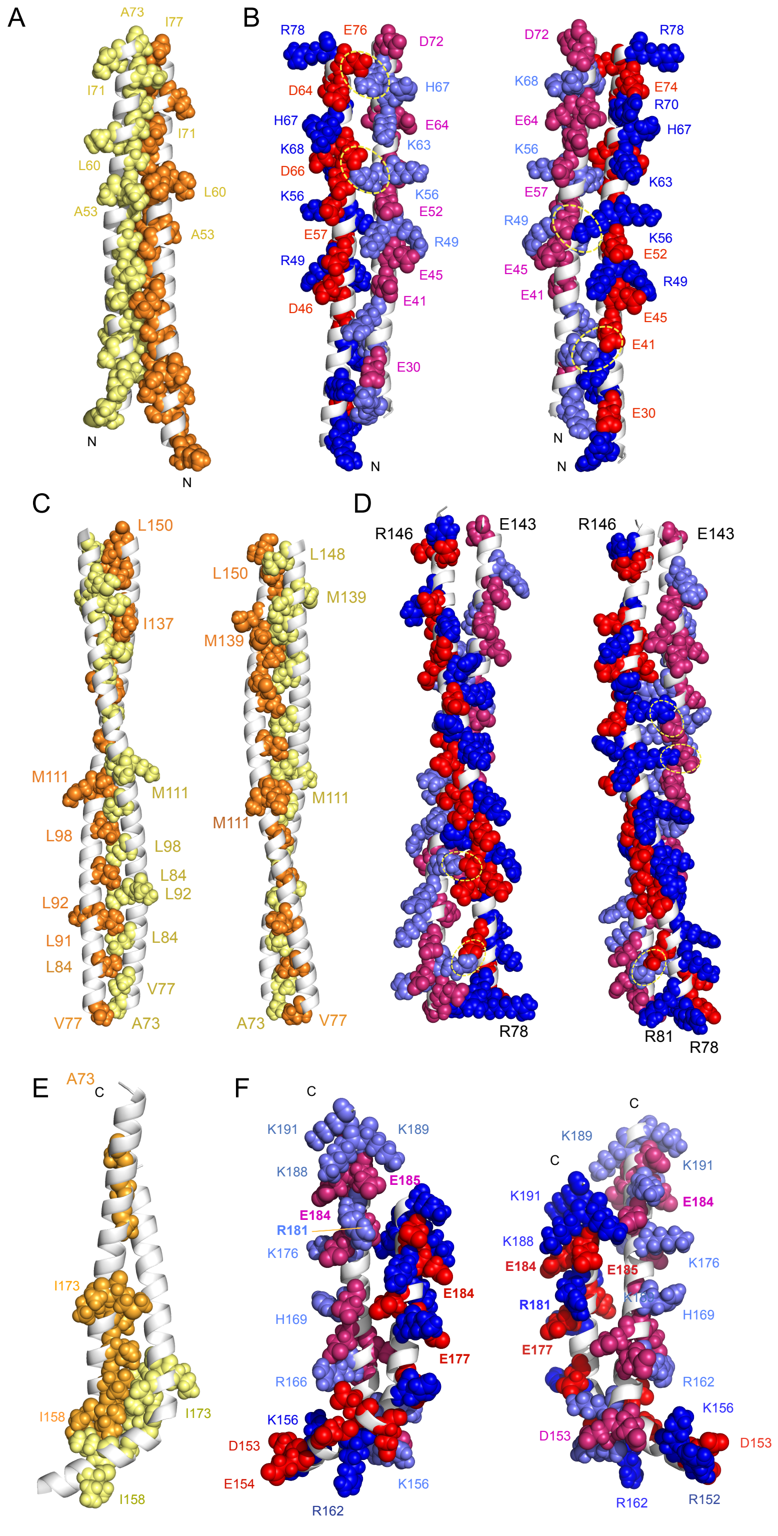

Figure S10: Hydrophobic (left) and electrostatic (right) interactions in the best model of the Nter-BsRNAseY dimer generated by AlphaFold. Two different views are shown in each case, except for A and E (only one view). A) and B) segment 22-78, C) and D) segment 78-146, E) and F) C-terminal segment (residues 152-192). The hydrophobic residues (A, I, L, M, V) are colored in orange (chain A) and yellow (chain B), the positively charged residues (K and R) are colored in dark blue (chain A) and light blue (Chain B) and the negatively charged residues (D and E) are colored in red (chain A) and pink (Chain B). The yellow dotted ovals show the electrostatic interactions.

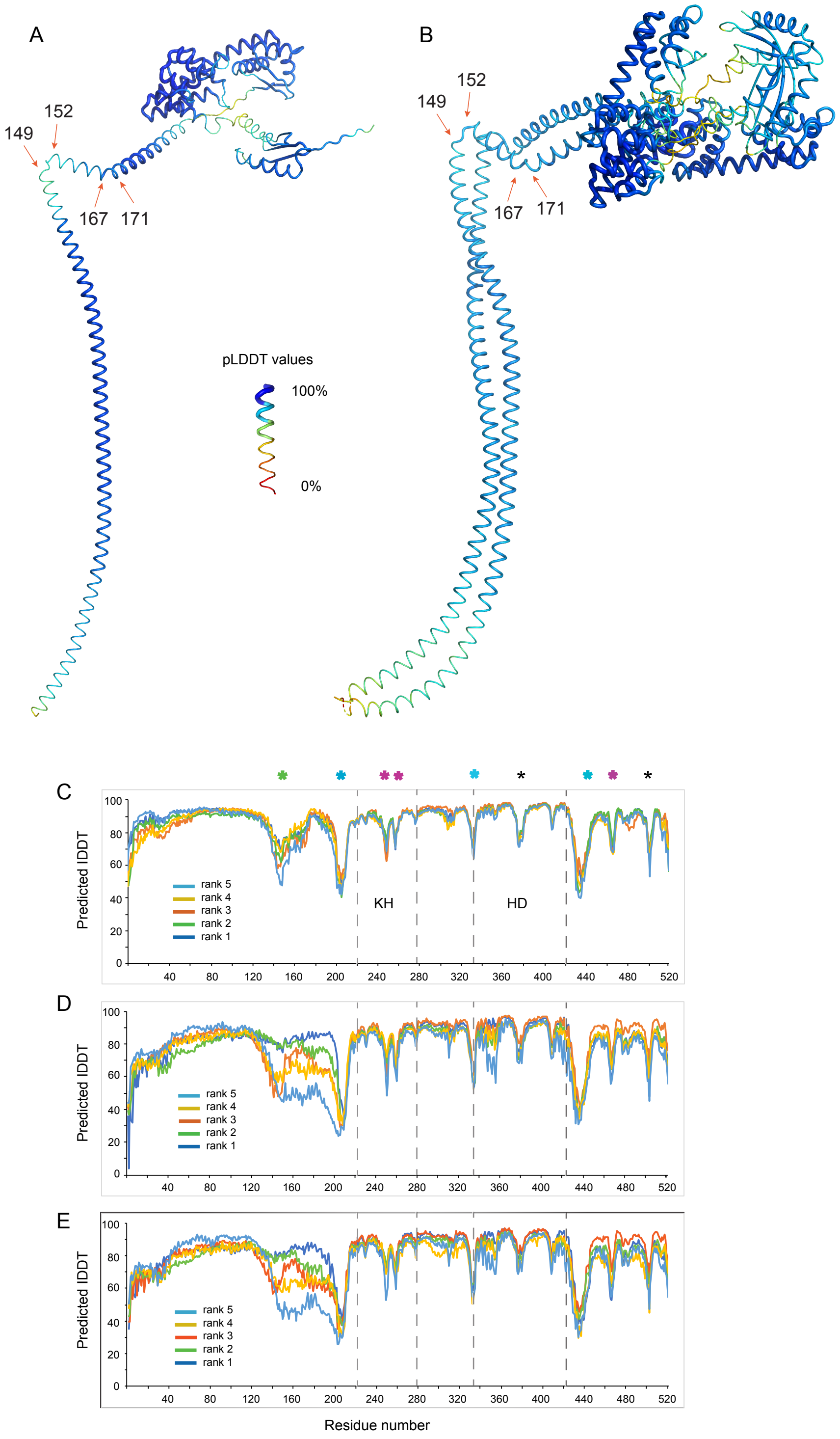

Figure S11: 3D models of the full-length *B. subtilis* RNase Y predicted by AlphaFold. The best AlphaFold models for the full-length *B. subtilis* RNase Y monomer (A) and dimer (B) are colored by their pLDDT values, from red (0%) to blue (100%). The residues at the edges of the turn (residues 148 to 154) and at the kink in the N-terminal helix (167-171) are highlighted as orange arrows. Per-residue confidence metrics for the five best AlphaFold models of the monomer (C) and of each monomer of the dimer (D and E). The vertical dashed lines separate the different domains of the full-length *B. subtilis* RNase Y. Stars at the top of the plot annotate the loops between β-strands (in pink), loops 203-210, 329-335, 430-437 linking the various domains of RNase Y (in blue), between α-helices (in black), and the turn in the coiled-coil domain (residues 150-151, in green).

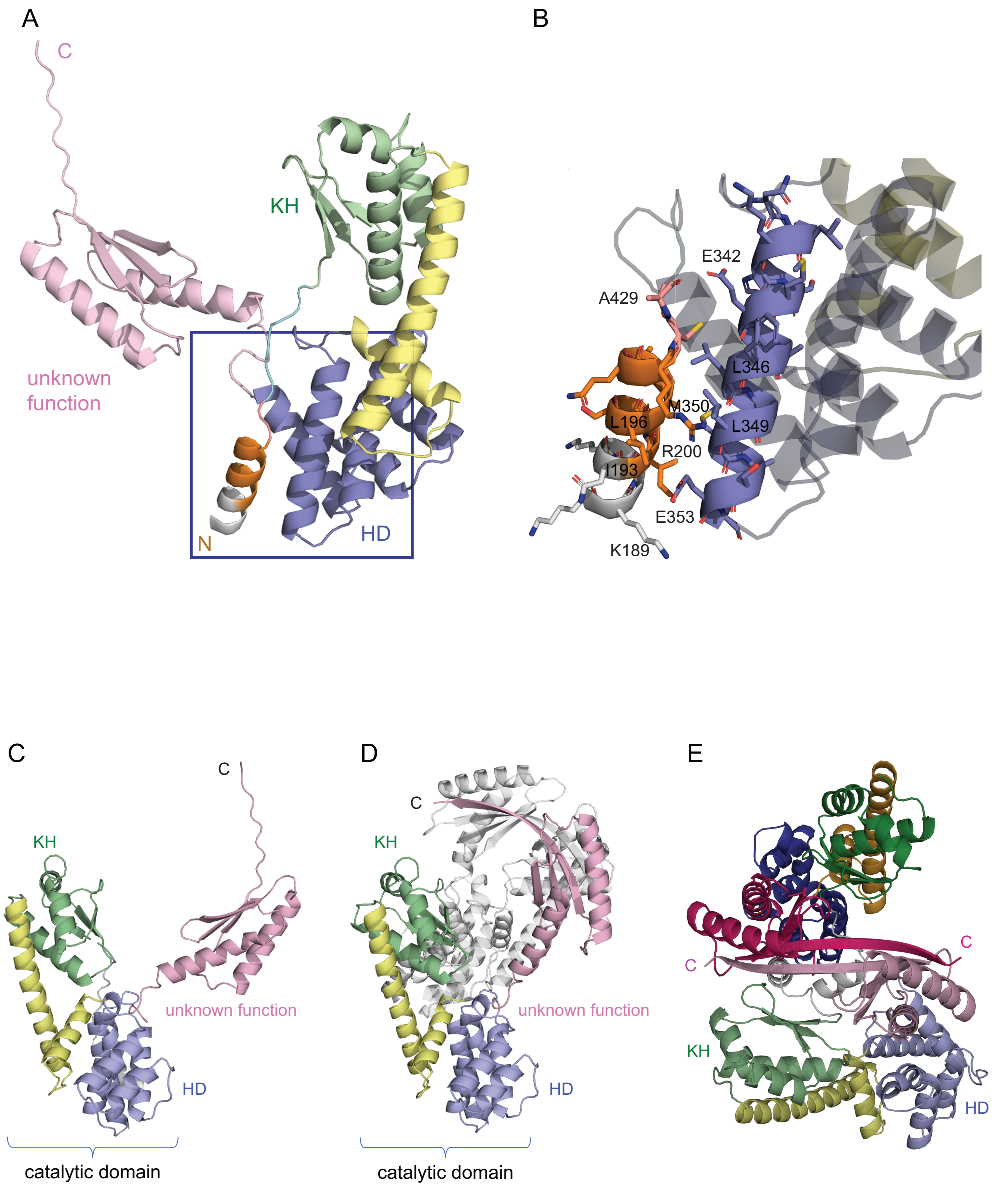

Figure S12. Best AlphaFold models for the C-terminal domain in the *B. subtilis* RNase Y monomer and dimer. The RNA binding domain (KH) (residues 211-276) is colored in green, residues 281 to 328 that form two α-helices in yellow, the HD domain (residues 336-429) in blue and the domain with unknown function (residues 438-520) in pink. (A) View of one monomer showing the possible interactions of the last α-helix of the N-terminal domain with the C-terminal domain. The last residues of the N-terminal domain (residues 188 to192 in white) form an extended helix with residues 193 to 202 (in orange) of the C-terminal domain. 'C' and 'N' indicate the position of the C and N terminus, respectively. (B) Enlarged view detailing the interactions between residues 193-197 from the N-terminal domain and residues 346-354 of the C-terminal domain. (C) Another view of the C-terminal domain in the best AlphaFold model of the monomer. (D) Same view of the C-terminal domain in the best AlphaFold model of the dimer. The second monomer is represented in grey. (E) View of the interaction surface in the dimer showing that the C-terminal region of unknown function is likely a dimerization domain. One chain is colored in light color and the second in dark color.

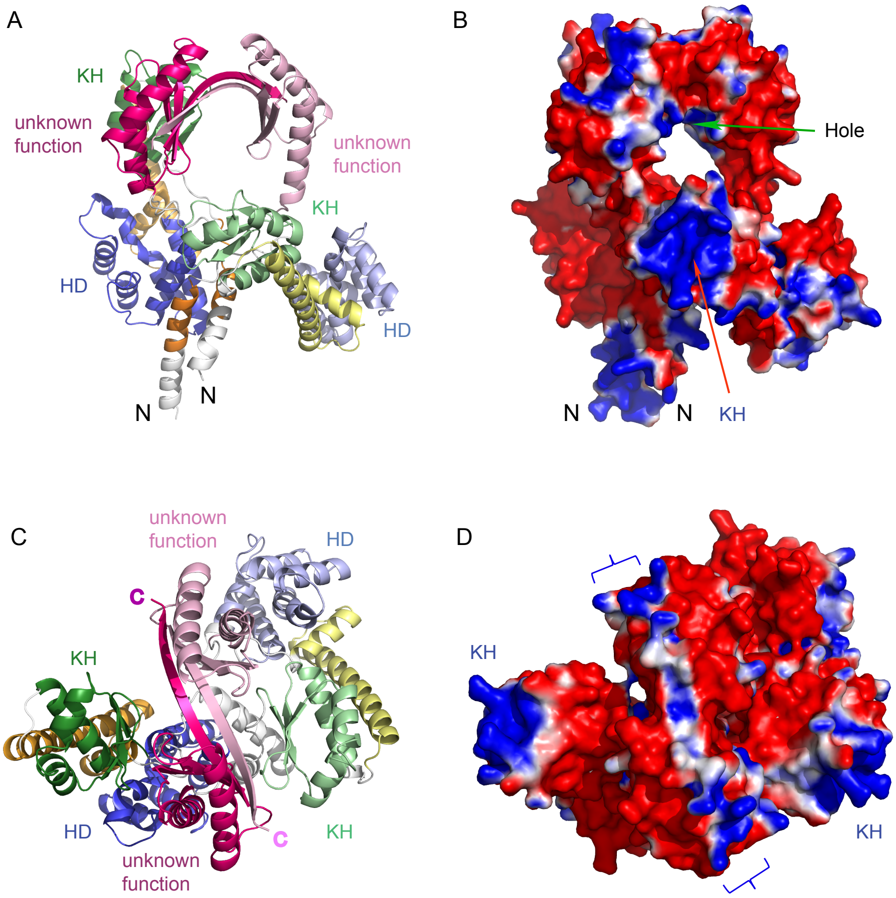

Figure S13: Dimeric interface of the C-terminal domain of the best AlphaFold model for the full-length *B. subtilis* RNase Y dimer. Two different orientations are shown (A) and (C), with their calculated electrostatic potential surface (B) and (D). The red and blue colors represent negative and positive charges, respectively. The red arrow highlights one of the KH domain and the green arrow the positively charged hole. The blue brackets frame the very C-terminal β-sheet of each monomer.

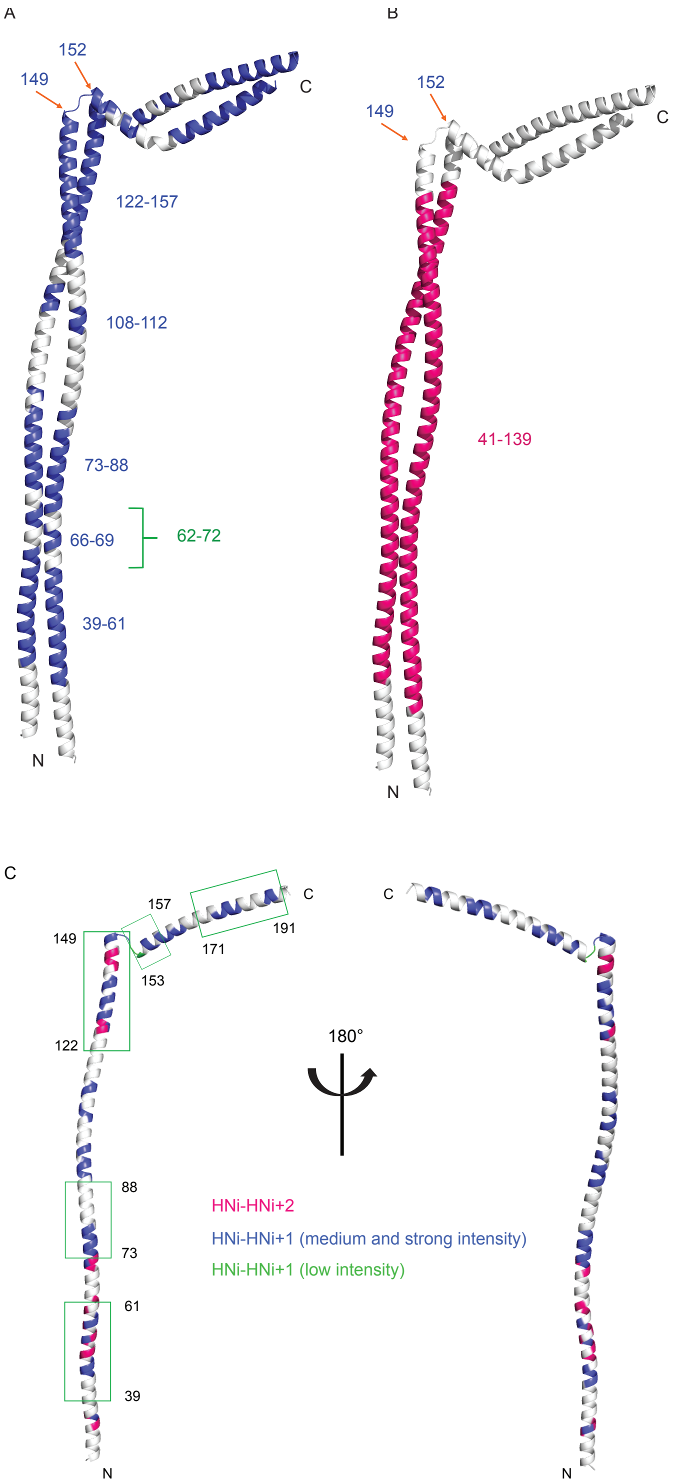

Figure S14: Comparison of the α-helices propensity of the Nter-BsRNaseY dimer, determined experimentally from the NMR chemical shifts or calculated with AlphaFold. (A) The α-helices with propensity (> 0.5) predicted by TALOS from the backbone NMR chemical shifts at 300 K are colored in blue. From the NMR data, it is not predicted that residues 89-121 would take part in an helical structure. (B) In pink: the best AlphaFold model of Nter-BsRNaseY showing only residues that possess pLDDT > 90%. The green bracket highlights the break in the supercoiled structure observed experimentally. (C) AlphaFold model of the monomer with the HN_i-HN_{i+1} cross-peaks that could be unambiguously assigned in blue (medium and strong intensity) or green (low intensity), and the HN_i-HNi_{+2} cross-peaks in red. The green rectangles indicate the positions of the helices predicted by TALOS with the highest scores.

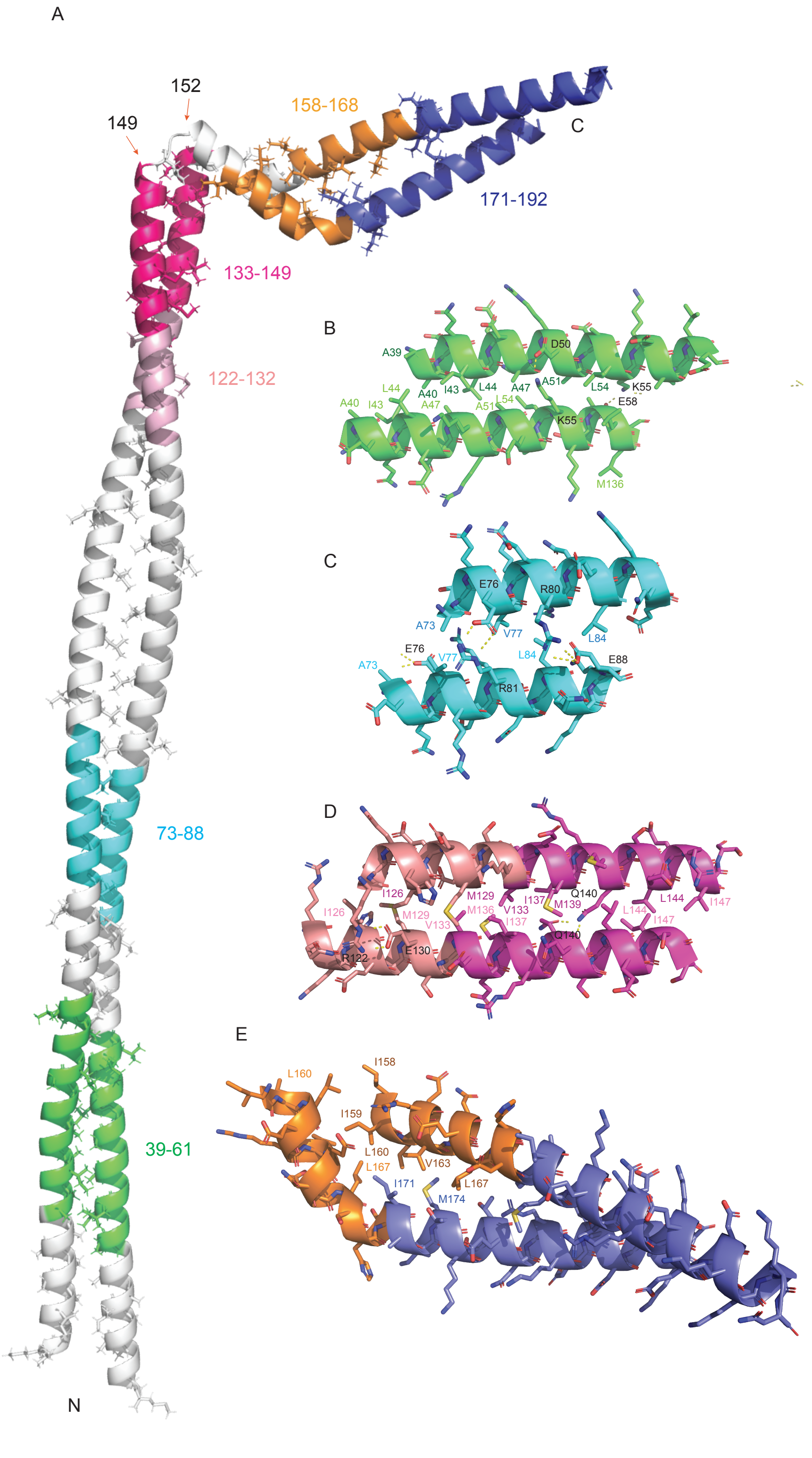

Figure S15: Comparison of the α-helices propensity determined experimentally from the backbone NMR chemical shifts at 300K using TALOS (Helices with $S^2 > 0.6$) and the AlphaFold Nter-BsRNaseY best dimer 3D model. (A) 3D model of the Nter-BsRNaseY dimer calculated by AlphaFold, with the α-helices predicted by TALOS highlighted in color: residues 39-61, green (B); residues 73-88, light blue (C); residues 122-132, light pink and residues 133-149 forming the most stable helix in pink (D). A break of the supercoil formed by the two helices occurs in the AlphaFold model around positions 62-72. (E) Residues 158-170 that undergo chemical exchange are shown in orange and residues 171-191 that have duplicated cross-peaks are in blue. The hydrophobic interactions are highlighted.

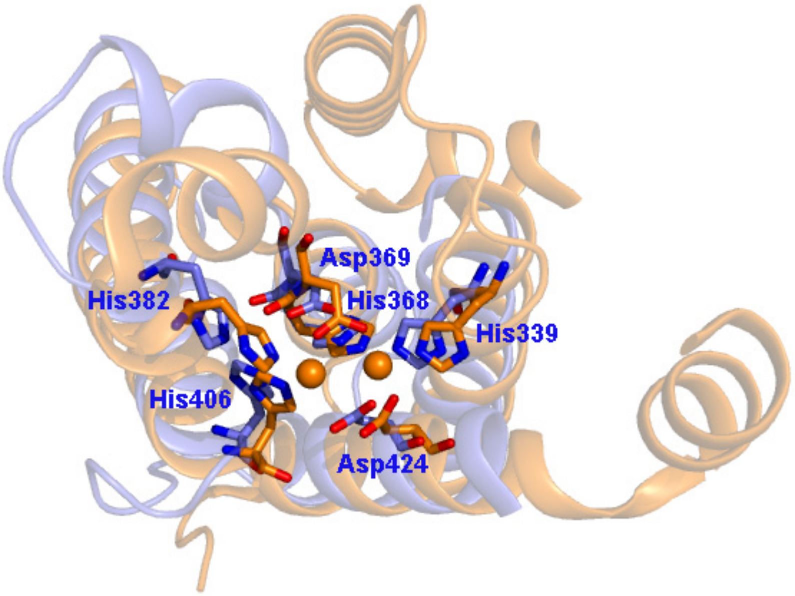

Figure S16: Potential binding mode of divalent metal ions to the HD domain of *B. subtilis* RNase Y. The HD domains of *B. subtilis* RNase Y (AlphaFold model in blue) and that of PDB code 2PQ7 (in orange) have been superimposed with the SUPER option in PYMOL (rmsd of 3.37 Å for 79 aligned Cαs). The Fe atoms in 2PQ7 are shown as orange spheres. The His/Aps residues that bind the Fe atoms in 2PQ7 (shown in orange stick representation) are conserved in *B. subtilis* RNase Y (His/Asp shown in blue stick representation and labeled in blue).

Table S1: Prediction of α-helices propensity using the α-helix prediction calculated by TALOS-N

helix	300K	303K	313K	S^2-TALOS-N 300K	S^2-TALOS-N 303K
α1	39-61	39-60	46-60	0.72	0.64
	66-69	66-67	66		
α2	73-88	73-85	73-78	0.70	0.62
	91-92	91	91		
	108-112	108-111	108-111		
α3	122-157	122-124<br>127-156	122-125<br>133-149<br>154-156	0.72<br>(133-149) 0.79	0.68<br>(133-149) 0.76
	161-163	161-163			
α4	171-191	176-190	181-186<br>189-191	0.73	0.66
% α helix	65	56	36		

S^2-TALOS-N, the estimated backbone order parameter S^2, is close to 1 for rigid bonds and 0 for flexible bonds. An α-helix is defined by a S^2 value above 0.69, whereas a value lower than 0.69 indicates high flexibility, which is typical for disordered segments.
